# Sequence Neighborhoods Enable Reliable Prediction of Pathogenic Mutations in Cancer Genomes

**DOI:** 10.3390/cancers13102366

**Published:** 2021-05-14

**Authors:** Shayantan Banerjee, Karthik Raman, Balaraman Ravindran

**Affiliations:** 1 Robert Bosch Centre for Data Science and Artificial Intelligence (RBCDSAI), Indian Institute of Technology (IIT) Madras, Chennai 600 036, India; bt16s001@smail.iitm.ac.in; 2 Initiative for Biological Systems Engineering, Indian Institute of Technology (IIT) Madras, Chennai 600 036, India; 3 Department of Biotechnology, Bhupat and Jyoti Mehta School of Biosciences, Indian Institute of Technology (IIT) Madras, Chennai 600 036, India; 4 Department of Computer Science and Engineering, Indian Institute of Technology (IIT) Madras, Chennai 600 036, India

**Keywords:** cancer driver mutations, context of mutations, neighborhood sequences, missense mutations, machine learning

## Abstract

**Simple Summary:**

Cancer is caused by the accumulation of somatic mutations, some of which are responsible for the disease’s progression (drivers) while others are functionally neutral (passengers). Although several methods have been developed to distinguish between the two classes of mutations, very few have concentrated on using the neighborhood nucleotide sequences as potential discrimination features. In this study, we show that driver mutations’ neighborhood is significantly different from that of passengers. We further develop a novel machine learning tool, NBDriver, which is highly efficient at identifying pathogenic variants from multiple independent test datasets. Efficient and accurate identification of novel pathogenic variants from sequenced cancer genomes would help facilitate more effective therapies tailored to patients’ mutational profiles.

**Abstract:**

Identifying cancer-causing mutations from sequenced cancer genomes hold much promise for targeted therapy and precision medicine. “Driver” mutations are primarily responsible for cancer progression, while “passengers” are functionally neutral. Although several computational approaches have been developed for distinguishing between driver and passenger mutations, very few have concentrated on using the raw nucleotide sequences surrounding a particular mutation as potential features for building predictive models. Using experimentally validated cancer mutation data in this study, we explored various string-based feature representation techniques to incorporate information on the neighborhood bases immediately 5′ and 3′ from each mutated position. Density estimation methods showed significant distributional differences between the neighborhood bases surrounding driver and passenger mutations. Binary classification models derived using repeated cross-validation experiments provided comparable performances across all window sizes. Integrating sequence features derived from raw nucleotide sequences with other genomic, structural, and evolutionary features resulted in the development of a pan-cancer mutation effect prediction tool, NBDriver, which was highly efficient in identifying pathogenic variants from five independent validation datasets. An ensemble predictor obtained by combining the predictions from NBDriver with three other commonly used driver prediction tools (FATHMM (cancer), CONDEL, and MutationTaster) significantly outperformed existing pan-cancer models in prioritizing a literature-curated list of driver and passenger mutations. Using the list of true positive mutation predictions derived from NBDriver, we identified a list of 138 known driver genes with functional evidence from various sources. Overall, our study underscores the efficacy of using raw nucleotide sequences as features to distinguish between driver and passenger mutations from sequenced cancer genomes.

## 1. Introduction

Cancer is caused due to the accumulation of somatic mutations during an individual’s lifetime [1]. These mutations arise due to both endogenous factors such as errors during DNA replication or exogenous factors such as substantial exposure to mutagens such as tobacco smoking, UV light, and radon gas [2,3,4]. These somatic mutations can be of different types, ranging from single-nucleotide variants (SNVs) to insertions and deletions of a few nucleotides, copy-number aberrations (CNAs), and large-scale rearrangements known as structural variants (SVs) [5]. With the advent of high-throughput sequencing, the identification of somatic mutations from sequenced cancer genomes has become more accessible. International cancer genomics projects have resulted in the development of large mutational databases such as the Catalogue of Somatic Mutations in Cancer (COSMIC) [6], the International Cancer Genome Consortium (ICGC) [7], and The Cancer Genome Atlas (TCGA) [8]. Several open-access resources to analyze and visualize large cancer genomics datasets, such as the cBio Cancer Genomics Portal [9] and the Database of Curated Mutations in cancer (DoCM) [10], have also been developed. These resources aggregate functionally relevant cancer variants from different studies and help researchers gain easy access to expert-curated lists of pathogenic somatic variants.

However, not all somatic mutations present in the cancer genome are equally responsible for developing the disease. A small fraction of somatic variants known as “driver mutations” provide a growth advantage and are positively selected for during cancer cell development [1]. On the other hand, “passenger mutations” provide no growth advantage and do not contribute to cancer progression [1]. Identifying the complete set of cancer-causing genes that harbor driver mutations, also known as driver genes, holds much promise for precision medicine, where a specific therapeutic intervention is tailored toward a patient’s mutational profile [11].

Distinguishing between driver and passenger mutations from sequenced cancer genomes is a non-trivial task. Doing so solely based on the substitution type (A > T, G > C, etc.) is very difficult. Hence, several computational methods that use several other factors to identify driver mutations have been developed over the years. Recurrence-based driver prioritization tools such as MutSigCV [12] and MuSiC [13] for single-nucleotide variants, and GISTIC2 [14] for copy-number aberrations, have been developed to identify variants that occur more than what is expected by chance, otherwise known as the “background mutation rate.” Other methods such as SIFT [15], PROVEAN [16], PolyPhen-2 [17], CHASM [18], and FATHMM [19] are based on predicting the functional impact of mutations on the protein encoded by the gene. Expert-curated databases such as the OncoKB database [20] contains information regarding the functional impact of over 3000 cancer-causing alterations belonging to over 400 genes. Pathway analysis-based tools such as NetBox [21] and HotNet [22] work by identifying mutations affecting large-scale gene regulatory or protein–protein interaction networks. Machine learning-based methods have also been recently developed to predict deleterious missense mutations [23,24,25,26,27,28].

Genome instability, demonstrated by a higher than average rate of substitution, insertion, and deletion of one or more nucleotides, is a hallmark of most cancer cells. There is a considerable variation in the rates of SNPs across the human genome. Sequence context plays a significant role in the variability of the substitutions rate as explained by the CpG dinucleotides, which exhibit an elevated C > T substitution rate by almost 15 folds relative to the average rate observed in mammals [29]. Mutational hotspots such as the CpG dinucleotides in breast and colorectal cancer [30] and TpC dinucleotides in lung cancer, melanoma, and ovarian cancer [31] are some examples of “signatures” that promote mutagenesis. There have been several efforts to use the sequence context to measure the human genome’s substitution rates. Aggarwala et al. [32] used SNPs’ local sequence context to explain the observed variability in substitution rates. Zhao et al. [33] studied the neighboring nucleotide biases and their effect on the mutational and evolutionary processes for over two million SNPs.

Recent studies have identified specific signatures or patterns of mutations in different cancer types that shed light on the underlying mechanisms responsible for cancer progression [34,35]. Alexandrov et al. [34] identified 21 distinct mutational signatures in human cancers by considering the substitution class and the sequence context immediately to the 3′ and 5′ of the mutated base. Several studies have demonstrated that certain factors such as tobacco smoking, UV light, or the inactivation of tumor suppressor genes involved in DNA repair can result in the development of mutational hotspots [31,34,36]. In particular, some of these studies [34,36] have shown that the characteristic nucleotide contexts surrounding passenger mutations indicate the underlying mutational processes active in the given tumor. Dietlin et al. [37] hypothesized that mutations occurring in nucleotide contexts that deviate from these characteristic passenger mutation contexts are functionally relevant and thus provide a signal in favor of tumor progression (also known as drivers). They further used these “unusual” nucleotide contexts as an indirect substitute for functional relevance and built probabilistic models to identify driver genes. Similarly, Agajanian et al. [38] integrated classical machine learning and deep learning approaches to model the surrounding nucleotide context to differentiate between driver and passenger mutations. 

In this study, our overall aim is to build models using machine learning and natural language processing techniques to differentiate between driver and passenger mutations solely based on the raw nucleotide context. Using missense mutation data with experimentally validated functional impacts compiled from various studies, we show that the underlying probability distributions of driver and passenger mutations’ neighborhoods are significantly different from one another. We extracted features from the neighborhood nucleotide sequences and built robust binary classification models to distinguish between the two classes of mutations. We achieved good classification performances during our repeated cross-validation experiments and against an independent hold-out set of literature-curated mutations. Integrating neighborhood features with other features such as protein physicochemical properties and evolutionary conservation scores significantly improved our algorithm’s overall predictive power in identifying pathogenic variants from five separate independent test sets and had comparable performances with some of the existing state-of-the-art mutation effect prediction tools. Overall, this study establishes that we can leverage efficient feature representation of the neighborhood sequences of cancer-causing mutations to differentiate between a known driver and passenger mutations with sufficient discriminative power.

## 2. Methods

### 2.1. Mutation Datasets for Building and Evaluating the Models

Our training data consisted of the list of missense mutations whose effects were determined from experimental assays and were compiled in the study conducted by Brown et al. [39]. In this study, missense mutations from 58 genes that were pan-cancer based were combined from five different datasets [40,41,42,43,44,45] (Table S1A). These mutations were presented as amino acid substitutions based on their protein coordinates (e.g., F595L, L597Q, etc.). Since we were interested in studying the effects of neighboring DNA nucleotide sequences, we mapped them to their corresponding genomic coordinates (gDNA) for further analysis. We used the publicly available TransVar web interface [46] for this purpose. The final training set was made up of 5265 single-nucleotide variants (4131 passengers and 1134 drivers).

For external validation, we collected somatic mutation data from five different sources. First, we considered a literature-curated list of 140 passengers and 849 driver mutations categorized based on functional evidence published by Martelotto et al. [45] as part of the benchmarking study to rank various mutation effect prediction algorithms.

Second, we used a subset of mutations published by the recently released Cancer Mutation Census. The Cancer Mutation Census (CMC) [6] is a recent undertaking that integrates all coding somatic mutation data from the COSMIC database and identifies variants driving different cancer types. It contains functional evidence obtained using both manual curation and computational predictions from multiple sources. We chose only single-nucleotide variants classified as missense and derived from the Cancer Gene Census (CGC)-classified list of tumor suppressor genes and oncogenes for our validation experiments. Based on the database’s various evidence criteria, we considered only mutations categorized as tier 1, 2, and 3 for our study. From this list, we further removed all overlapping mutations with our training set and derived a final set of 277 mutations for further analysis.

The Catalog of Validated Oncogenic Mutations from the Cancer Genome Interpreter [35] database contains a high-confidence list of pathogenic alterations compiled from several sources such as the DoCM [10], ClinVar [47], OncoKB [20], and the Cancer Biomarkers Database [35]. We extracted only missense somatic mutations flagged as “cancer” for our validation experiments. After removing all overlapping mutations with our training set, we obtained a final list of 1628 driver mutations. This constituted our third validation set.

The fourth validation dataset consisted of the list of top 50 hotspot mutations reported in the comprehensive study performed by Rheinbay et al. [48]. In this study, mutation data was accumulated from the Pan-Cancer Analysis of Whole Genomes (PCAWG) consortium and involved analyzing more than 2700 cancer genomes derived from more than 2500 patients. A total of 33 coding missense mutations from five well-known cancer genes: TP53, PIK3CA, NRAS, KRAS, IDH1, were extracted from this study.

Mao et al. [27] published mutation datasets to judge the driver prediction tool’s performance (CanDrA) in predicting rare driver mutations. They were constructed using the following criteria:

1. Glioblastoma (GBM) and Ovarian Cancer (OVC) mutations reported in the COSMIC database only once;
2. The reported mutations had no other mutations within 3bp of their position and were not part of either the training or test datasets for building the machine learning model (CanDrA).

We used the same datasets to judge our model’s ability to predict rare driver mutations based solely on the neighborhood sequences. After removing all overlapping mutations with the training set, we obtained 34 GBM mutations and 38 OVC mutations. A summary of all the mutational datasets used in our study is available in Table S1B. All our predictions are derived using the forward strand and were based on the GRCh37 (ENSEMBL release 87) build of the human genome.

### 2.2. Feature Extraction

#### 2.2.1. Sequence-Based Features

**Figure 1:**
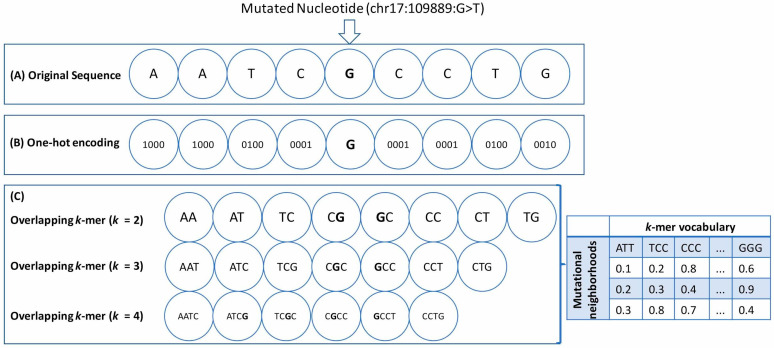
Neighborhood feature representations: A diagram representing the features derived from the neighborhood nucleotide sequences of the point mutations for an arbitrary window size of 4 is shown here. The mutated position is represented as a triplet (chromosome: position: substitution type). (**A**) The original sequence is represented here with the mutated nucleotide (ch17:109889:G > T) in bold. (**B**) One-hot encoding was used to derive the 4-bit binary one-hot encoded vector for each nucleotide. (**C**) Overlapping *k*-mers of sizes 2, 3, and 4 have been represented here. In this case, the neighborhood features also include the wild-type nucleotide at the mutated position. The overlapping *k*-mers were encoded into a numerical format using the Count Vectorizer and the TF-IDF Vectorizer, and the resulting word matrix was derived. The samples (or individual neighborhoods) are represented as rows, and the *k*-mers are represented as columns. The chromosome number and the substitution type (A > T, G > C, etc.) were included as additional features for both types of feature representation.

We used the raw nucleotide sequences surrounding a mutation as features for our analysis. Each unique mutation was represented as a triplet (chromosome, position, type) where “type” refers to one of the 12 types of point substitution (A > T, A > G, A > C, T > A, T > G, T > C, G > A, G > C, G > T, C > T, C > A, C > G). We then extracted the surrounding raw nucleotide sequences from the reference genome for a given mutation position using the bedtools Consolas command. The “window size” for a particular mutation captures the number of nucleotides upstream and downstream from the mutated position. Hence, considering all possible window sizes between 1 and 10, including the wild-type nucleotide at the mutated position, we obtained nucleotide strings of length 3, 5, 7, 9, 11, 13, 15, 17, 19, and 21, respectively. We also considered the chromosome number and the type of point substitution as features for our analysis. Now, for particular window size, to map the nucleotide strings to a numerical format, we used the following two widely used feature transformation approaches (Figure 1A–C):

1. **One-hot encoding** (**OHE**)**:** Each neighboring nucleotide was represented as a binary vector of size 4 containing all zero values except the nucleotide index, marked as 1 (Figure 1A,B). Thus “A” was encoded as 〈1,0,0,0〉, “G” as 〈0,1,0,0〉, and so on.
  This particular feature representation resulted in a feature space of size 8n+2, where n=1,2,3…10 represents the window sizes. We used the pandas get_dummies() to perform this task.
2. **Overlapping *k*-mers:** In this type of feature representation, the neighboring nucleotide string sequences for a given window size were represented as overlapping *k*-mers of lengths 2, 3, and 4 (Figure 1C). For instance, an arbitrary sequence of window size 3 {ATT**T**GGA}, where ‘**T**’ is the wild-type base at the mutated position, can be decomposed into overlapping ***k***-mers of size 2 {AT, TT, T**T**, **T**G, GG, GA}, 3 {ATT, TT**T**, T**T**G, **T**GG, GGA}, and 4 {ATT**T**, TT**T**G, T**T**GG, TGGA}, respectively.

To map these overlapping *k*-mers to a numerical format, we applied two commonly used encoding techniques known as Count Vectorizer (CV) and TF-IDF Vectorizer (TF). The Count Vectorizer returns a vector encoding whose length is equal to that of the vocabulary (total number of unique *k*-mers in the dataset) and contains an integer count for the number of times a given *k*-mer has appeared in our dataset.

A term frequency-inverse document frequency (TF-IDF) vectorizer assigns scores to each *k*-mer based on (i) how often the given *k*-mer appears in the dataset and (ii) how much information the given *k*-mer provides, i.e., whether it is common or rare in our dataset. Mathematically, for a given term *i* present in a document *j*, the TF-IDF score $tf_{i,j}$ is given by

$$tf_{i,j}=freq_{i,j}\times log\frac{N}{d_{i}}$$

where $freq_{i,j}$ is the number of occurrences of *i* in *j*, $d_{i}$ is the number of documents containing *i*, and *N* is the total number of documents. These techniques were implemented in Python using the *feature_extraction* module from scikit-learn. The final processed training set used to build the machine learning models was represented as a matrix of size mn, where *m* is the total number of coding point mutations and *n* is the size of the vocabulary. The matrix entries were the TF-IDF or the Count Vectorizer scores. The number of one-hot encoded features, *k*-mers, and the size of the vocabulary possible for each window size is shown in Table S2B.

#### 2.2.2. Descriptive Genomic Features

In addition to the neighborhood features, a set of 27 features (Table S2A) previously used to train the cancer-specific missense mutation annotation tool, CanDrA [27], were extracted from the following three data portals: CHASM’s SNVBOX [18], Mutation Assessor [25] and ANNOVAR [49]. While deriving the final binary classifier, NBDriver, we augmented the neighborhood sequence features derived using the one-hot encoding, Count Vectorizer, and TF-IDF-based feature representation with these descriptive genomic features. Among them were conservation scores (such as GERP scores, HMMPHC scores, and others), amino acid substitution features (such as PREDRSAE, PredBFactorS, and others), exon features (such as ExonSnpDensity, ExonConservation, and others), features indicative of protein domain knowledge (such as UniprotDOM_PostModEnz, UniprotREGIONS, and others) and functional impact scores computed by algorithms such as VEST [23] and CHASM [18]. A tiny fraction (0.1%) of the UniProtKB annotations were not available from the SNVBOX database for our training data. We used the *k*-nearest neighbors-based imputation technique to substitute the missing features with those of the same gene’s nearest mutations. Our external validation datasets were free from any missing information.

### 2.3. Density Estimation

A kernel density estimator (or KDE) takes an *n*-dimensional dataset as an input and outputs an estimate of the underlying *n*-dimensional probability distribution. A Gaussian KDE uses a mixture of *n*-dimensional Gaussian probability distributions to represent the density being estimated. It essentially tries to center one Gaussian component per data point, resulting in a non-parametric estimation of the density. One of the hyperparameters for a kernel density estimator is the bandwidth, which controls the kernel’s size at each data point, thereby affecting the “smoothness” of the resulting curve. We estimated the underlying probability distributions for the driver and passenger neighborhoods using a Gaussian kernel density estimator.

The entire process’s schematic workflow for a single run of the kernel density estimation experiment is shown in Figure S2A–F. First, we randomly selected, with replacement, an equal number (*n*) of driver and passenger mutations from our training data for a single run of the kernel density estimation algorithm and particular window size (Figure S2A). Then, we tuned the bandwidth hyperparameter for each class of mutations using a 5-fold cross-validation approach and used the best parameters to derive the kernel density estimates (Figure S2B). Finally, we used the Jensen–Shannon (JS) distance metric to calculate the similarity between the two class-wise density estimates (Figure S2C). The JS distance between two probability distributions is based on the Kullback–Leibler (KL) divergence, but unlike KL divergence, it is bounded and symmetric. For two probability vectors, *p* and *q*, it is given by,

$$JS=\frac{1}{2}\sqrt{D\left(p∥m\right)+D\left(q∥m\right)}$$

where $m=\frac{1}{2}\left(p+q\right)$, and *D* is the KL divergence. The significance of the estimated distances between the probability estimates was calculated using a randomized bootstrapping approach. Specifically, we randomly sampled with replacement twice the number (*2n*) of mutations from the same training set, irrespective of the labels. We then split the dataset in half, randomly assigning each half to driver and passenger mutations, respectively (Figure S2D). This was followed by a similar process of tuning the hyperparameters and deriving the class-wise density estimates (Figure S2E). Finally, we reported the JS distance between the density estimates (Figure S2F).

We experimented with the following seven different neighborhood-based feature representations:

- One-hot encoding;
- Count Vectorizer (*k*-mer sizes of 2, 3 and 4);
- TF-IDF Vectorizer (*k*-mer sizes of 2, 3 and 4).

The aforementioned KDE estimation experiments were repeated 30 times for all possible window sizes between 1 and 10 and all seven feature representations. Next, the best median JS distance estimate from the original experiments was reported for the given window size. The percentage of runs of the randomized experiments for which the estimated distance was greater than the original estimate was reported as the *p*-value. The KernelDensity() function from the scikit-learn *neighbors* module was used to derive the density estimates and jensenshannon() from the scipy *spatial.distance* submodule was used to calculate the distance metric.

### 2.4. Classification Models

We implemented three classifiers: the random forest (RF) classifier, the extra trees (ET) classifier (extreme random forests), and the generative KDE classifier to build our binary classification models. The overall approach for the KDE-based classification was as follows (Figure S1A):

1. The dataset was split using the cross-validation strategy;
2. The training data was then split by label (driver/passenger);
3. We fit a generative model for each class using the kernel density estimation method as described in the previous section. This provided us the likelihood that P(x|passenger) and P(x|driver), respectively, for a particular data point *x;*
4. Next, the class prior, given by the number of examples of each class, or, P(driver) and P(passenger) was calculated;
5. Now, for a test data point *x*, the posterior probability was given by P(driver|x)∝P(x|driver)P(driver) and P(passenger|x)∝P(x|passenger)P(passenger). The label that maximized the posterior probabilities was the one assigned to *x*.

In contrast, both the tree-based classifiers are discriminative. They are composed of a large collection of decision trees where the final output is derived by combining every single tree’s predictions by a majority voting scheme. The main difference between the two tree-based classifiers lies in selecting splits or cut points to split the individual nodes. Random forest chooses an optimal split for each feature under consideration, whereas extra trees choose it randomly. All the classification models were written using the predefined functions available in the *scikit-learn (v. 0.22)* [50] module.

### 2.5. Model Selection and Tuning

#### Repeated Cross-Validation Experiments

Owing to the relatively smaller sample size (5265 mutations) of the training set of mutations, we adopted a repeated 10-fold cross-validation approach to building our model. First, we split the dataset into ten equal subsets in a stratified fashion. Splitting the dataset in a stratified fashion maintains the same proportion of mutations in each class as observed in the original data. Nine of the ten subsets were combined into one training set (Figure S1A). In each training phase, we performed feature selection using the extra trees classifier, cross-validated grid search-based parameter tuning, training the classifiers using the best parameters, and obtaining the corresponding prediction scores on the hold-out test set (Figure S1B). For a given window size, we experimented with a total of seven feature representations (one-hot encoding, count vectorizer (*k*-mer size = 2, 3 and 4), TF-IDF vectorizer (*k*-mer size = 2, 3, and 4), and three binary classifiers (random forests, extra trees, and kernel density estimation)). So overall, we had 21 distinct feature-classifier pairs.

We ran the 10-fold cross-validation experiments (Figure S1A,B) three times for each such pair, thereby obtaining 30 values for each classification metric: sensitivity, specificity, AUROC, and MCC. The top five feature-classifier combinations in terms of the overall median value and the 95% CI for each of the above metrics were reported. To study the variation in classification performances with the addition of more nucleotides (or increase in window size), we repeated the Wilcoxon signed-rank test on the best performance metrics for all 45 pairs of window sizes (x,y), where x<y and (x,y)∈[1,2,…,10]. The ci() from the *gmodels* package [51] in R was used to calculate the 95% CIs for the various classification metrics.

### 2.6. Derivation of the Binary Classification Model to Distinguish between Driver and Passenger Mutations

To derive the final machine learning model, NBDriver, all overlapping mutations between the training set Brown et al., and the validation set Martelotto et al., were discarded, and the classifier was retrained on the reduced train set (4549 mutations: 544 drivers and 4005 passengers). The set of 989 mutations published by Martelotto et al. [45] formed our independent test set. Due to the inherent imbalance in the dataset, we implemented an undersampling technique known as repeated edited nearest neighbors [52] to downsize the majority class and consequently obtain a balanced dataset for subsequent training.

Predictions were obtained using two separate feature sets: (1) only neighborhood features based on the raw nucleotide sequences (or the neighborhood-only model) and (2) neighborhood features plus the descriptive genomic features (or NBDriver). In addition to random forests, extra trees, and the KDE classifier, we also experimented with a fourth classifier: a linear kernel SVM to obtain these predictions. Various combinations of these classifiers were implemented as ensemble models using the VotingClassifier() of the *ensemble* module in *scikit-learn*.

### 2.7. Feature Selection

We adopted an impurity-based feature selection technique for feature selection using the extra trees classifier to derive a ranked list of the top predictive features for our analysis. For the repeated cross-validation experiments, the features that were within the top 30 percentile of the most important features were selected and subsequently used to train our models. However, for deriving NBDriver, which was trained using a combination of both neighborhood sequence features and descriptive genomic features, we built several classification models based on the top *n* (*n* = 20, 30, 40, 50, 60) features. We chose the one that provided the best overall classification performance.

The TF-IDF and Count Vectorizer scores, used as features for our analysis, were implemented using the *feature_extraction* module in *scikit-learn*. In both cases, a new vocabulary dictionary of all the *k*-mers was first learned from the training data using the fit_transform() routine, and the corresponding term-document matrix was returned. Using this vocabulary, the scores of the *k*-mers from the test data were obtained using the transform() routine and were subsequently used in our analysis.

### 2.8. Hyperparameter Tuning and Classifier Threshold Selection

Hyperparameter tuning was performed using a cross-validation-based grid search technique over a parameter grid. The GridSearchCV() from the *model_selection* module in *scikit-learn* was used for this purpose. To further fine-tune the classifiers, we experimented with various classification thresholds from 0 to 1 with step sizes 0.001 and chose the one that provided the best areas under the ROC curve (AUROC). For an imbalanced classification problem, using the default threshold of 0.5 is not a viable option and often results in the incorrect prediction of the minority class examples.

### 2.9. Performance Metrics

For the repeated cross-validation experiments, we assessed our classifiers’ performance using four commonly used performance metrics: sensitivity, specificity, Matthews correlation coefficient (*MCC*), and area under the ROC curve (AUROC). Matthews correlation coefficient is a balanced metric and is very useful in imbalanced classification problems. It is bounded between −1 and 1, with −1 representing perfect misclassification, 0 representing average classification, and +1 representing ideal classification. It is given by the following expression:

$$MCC=\frac{TP\times TN-FP\times FN}{\sqrt{\left(TP+FP\right)\left(TP+FN\right)\left(TN+FN\right)\left(TN+FP\right)}}$$

where *TP* stands for true positives, *TN*, true negatives, *FP*, false positives, and FN, false negatives. *MCC* is a more robust alternative to accuracy and F1-score that can sometimes show overoptimistic classification performance for imbalanced data and was therefore not used for the analysis.

After deriving the binary classifier, we used additional classification performance metrics outlined by Martelotto et al. to compare our algorithm’s performance with other state-of-the-art mutation effect prediction tools. They were positive predictive value (PPV), negative predictive value (NPV), and a composite score, defined as the sum of sensitivity, specificity, PPV, and NPV.

### 2.10. Comparison with Other Pan-Cancer Mutation Effect Predictors

Similar to the benchmarking study conducted by Martelotto et al., we compared the generated binary classifiers with nine pan-cancer mutation effect prediction tools: MutationTaster [53], FATHMM (cancer) [19], Condel [26], FATHMM (missense) [19], PROVEAN (v1.1.3) [16], SIFT (Ensemble 66) [54], Polyphen2 [17], Mutation Assessor [25] and VEST [23] using the set of 989 literature-curated mutations. We used the prediction labels for each of these predictors based on predefined score cutoffs published as part of the Martelotto et al. [45] study. Two new prediction algorithms (CHASMplus (pan-cancer) [24] and CanDrA+ (Cancer-in general) [27]) were also added to the list, and the score cutoffs were decided in the following manner.

For CHASMplus, we tested all possible thresholds between 0 and 1 with step sizes of 0.01 and chose the corresponding threshold with the highest composite score due to the absence of a default threshold. All mutations with predicted scores greater than this optimal threshold were labeled as drivers and vice versa. For CanDrA+, we used the default prediction categories [27]. Predictions for CHASMplus and CanDrA+ were obtained from the OpenCRAVAT web server [55] and executable packages published by Mao et al. [27]. Two ensemble techniques were used to combine the outputs produced by different mutation effect predictors. First, we implemented a majority voting rule, also known as “hard voting,” where the class label with the maximum votes was chosen as the output. Second, we adopted a technique similar to Martelotto et al. [45], considering only the top four mutation effect predictors, and we generated different combinations by using *n* (*n* = 2, 3, 4) single predictors at a time. A given mutation predicted by this ensemble was considered a driver if at least *p* (*p* = 1, 2, 3, 4) predictors called it a driver for all combinations of *n* and *p*.

While comparing any two machine learning algorithms in this study by deriving the significance of the difference between any two classification metrics, we adopted the same strategy as Martelotto et al. Briefly, we derived the 95% CI for each of these classification metrics by repeated sampling with replacement with 1000 iterations. If the generated CI’s touched or there was no overlap, the difference was considered significant (p<0.05) based on the results of the analysis performed by Ng et al. [56]. The entire process’s schematic workflow for deriving NBDriver is shown in Figure S5.

## 3. Results

This study reports a pan-cancer machine learning tool, NBDriver, which uses neighborhood sequences as features to discriminate missense mutations as either drivers or passengers. Our key results are three-fold. First, we use generative models to derive the distances between the neighborhood sequences’ underlying probability estimates for the two mutation classes. Then, we build robust classification models using repeated cross-validation experiments to derive the median values of the metrics designed to estimate the classification performances. Finally, we demonstrate our models’ ability to predict unseen coding mutations from independent test datasets derived from large mutational databases.

### 3.1. Neighborhood Sequences of Driver and Passenger Mutations Show Markedly Different Distributions

**Figure 2:**
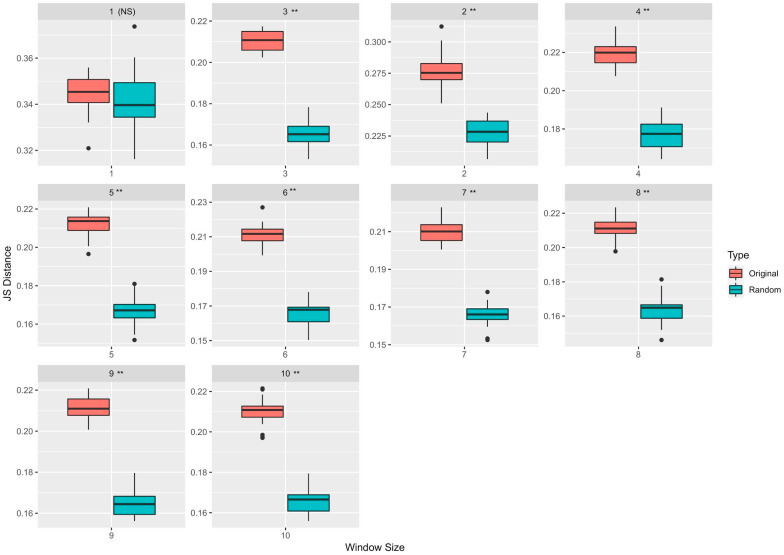
KDE estimation results: Variation in JS distances between the estimated densities for every window size between 1 and 10 is shown in this figure. All 5265 mutations from the original study were used here. Two types of boxplots, one for the original and another for the randomized experiments, have been shown here along with the *p*-values, which approximates the probability that the original median distance can be obtained by chance. Except for window 1, all other window sizes had a significant (** p<0.05) difference between the original and the randomized JS distances. (NS = Not Significant).

We estimated the driver and passenger neighborhood sequences’ underlying probability distributions using kernel density estimation. We computed the Jensen–Shannon (JS) distance metric to understand how “distinguishable” they are from one another. The JS metric is bounded between 0 (maximally similar) and 1 (maximally dissimilar). Table 1 shows the feature representations with the maximum median JS distances from the KDE experiments for various window sizes. We observed that, for the Brown et al. dataset [39], the maximum significant (p<0.05) median JS distance between passenger and driver neighborhood distributions, calculated across 30 runs of bootstrapping experiments, was 0.275 (for a window size of 2). However, there was no significant difference in terms of the median JS estimates calculated for window sizes 5 to 10 (Mann–Whitney U test; *p* < 0.05) using the different feature representations. Figure 2 shows the variation in the JS distances between the original and the randomized KDE experiments for window sizes between 1 and 10. As evident from Figure 2, except for window size 1, all other window sizes had a significant JS distance value (p<0.05).

Out of the seven different feature representations, we reported the ones that provided the maximum median JS distance. From Table 1, we observed that a TF-IDF Vectorizer with *k*-mer sizes 2, 3, and 4 was the preferred form of feature representation for six window sizes (1, 4, 6, 8, 9, and 10). In contrast, a Count Vectorizer with *k*-mer sizes 2 and 3 was chosen for three window sizes (3, 5, and 7). However, the only exception was for a window size of 2, where the one-hot encoding-based feature representation technique provided the maximum median JS distance. These results indicated that both the Count Vectorizer and the TF-IDF Vectorizer-based feature representation were consistent at delineating the distributions’ differences between the driver and passenger neighborhoods. Extended results from the KDE experiments containing the ranked median JS estimates for all seven different feature representations and their corresponding statistical significance are shown in Table S3C,D, respectively. From this table, we observed that there was a statistically significant (Mann–Whitney U test; *p* < 0.05) difference between the maximum overall median JS distance of 0.275 (window size = 2) obtained using the TF-IDF based feature representation with a *k*-mer size of 2 and the second best median JS distance of 0.255 (window size = 2), obtained using a Count Vectorizer with a *k*-mer size of 2. However, there was no significant difference between the minimum overall median JS distance estimate of 0.211, derived for window sizes 5 (using CV (*k* = 2, 3) and TF (*k* = 2, 3, and 4)), 7 and 8 (using CV (*k* = 2, 3 and 4) and TF (*k* = 3, 4)) and 9 and 10 (using CV (*k* = 2, 3) and TF (*k* = 2, 3 and 4)).

### 3.2. Repeated Cross-Validation Using Only Neighborhood Features Generates Robust Classification Models

**Figure 3:**
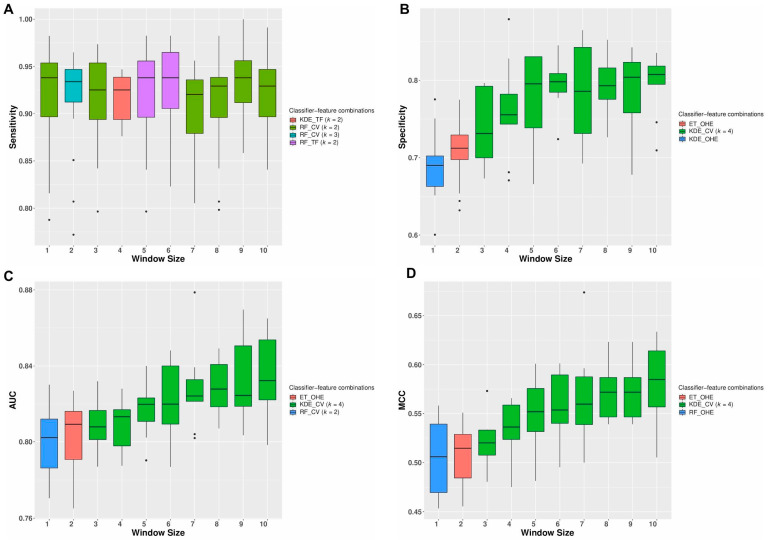
Repeated cross-validation results: The variation in the classification performances with different window sizes for the top feature-classifier combinations obtained during the repeated cross-validation experiments using the initial training set of 5265 mutations is shown in this figure. For each window size, the best results obtained using the different classifier-feature combinations in terms of (**A**) sensitivity, (**B**) specificity, (**C**) AUC, and (**D**) MCC are displayed.

The top five feature-classifier combinations in terms of the four classification metrics (sensitivity, specificity, AUROC, and MCC) calculated from the repeated cross-validation experiments using only the neighborhood sequences as features are shown in the Table S3A. From these results, we observed that the best median sensitivity of 0.938 (95%CI 0.929–0.942) was obtained using features derived from both the TF-IDF Vectorizer and the Count Vectorizer and subsequent training using a random forest classifier for window sizes 1, 5, 6, and 9. However, the best median specificity of 0.807 (95%CI 0.798–0.809), AUC of 0.832 (95% CI 0.826–0.841), and MCC of 0.584 (95% CI 0.564–0.594) were obtained using a Count Vectorizer-based feature representation trained using a KDE classifier for a window size of 10. The variation in the classification performances for the top three feature-classifier combinations obtained for different window sizes during the repeated cross-validation experiments using the initial training set of 5265 mutations is shown in Figure S6A–D. Classification metrics such as AUC and MCC are used to measure the quality of binary classifications. Except for window sizes 1 and 2, both the TF-IDF Vectorizer and Count Vectorizer performed consistently well and were among the top three feature representations in terms of the overall AUC and MCC, indicating that these feature representation techniques were the most efficient separating the two classes of mutations. Similarly, the best results obtained using the different feature-classifier combinations for various window sizes are summarized in Figure 3A–D. From this figure, we see that except for window sizes 1 and 2, a Count Vectorizer derived using a *k*-mer size of four provided the maximum median AUC, Specificity, and MCC. However, the maximum median sensitivities were obtained for all window sizes using both the Count Vectorizer and TF-IDF Vectorizer-based feature representation technique. For window sizes 1 and 2, a one-hot encoding-based feature representation provided the best MCC.

The variation in the classification performances with the window size increase is shown in Table S3B. From this table, we observed that out of the 45 unique pairs of window sizes (Methods: Repeated cross-validation experiments), 27 had a significant (p<0.05; Wilcoxon signed-rank test) increase in specificity and AUC while 31 had a significant (p<0.05; Wilcoxon signed-rank test) increase in MCC with the addition of more nucleotides. However, for sensitivity, a significant increase was observed only when the window size was increased from 4 to 9 and 7 to 9, respectively. These results indicated that adding more nucleotides to a particular window does not always guarantee an increase in the classifier’s performance in distinguishing between driver and passenger mutations.

### 3.3. Classification Models Provide Performances Comparable with Other State-of-the-Art Mutation Effect Predictors

Using only the neighborhood nucleotide sequences as features, the best results (Table 2; Table S5B) on the independent test set [45], was obtained using an extra trees classifier. This “neighborhood-only model” was trained on sequence features extracted using the count vectorizer technique on a window size of 10.

We trained NBDriver by combining the neighborhood features and the descriptive genomic features. Out of the various classifiers implemented, an ensemble model consisting of a linear kernel SVM and a KDE classifier provided the best results (Table 2; Table S5B) that consisted of 791 true positive, 50 false positive, 58 false negative, and 90 true negative mutations. Compared to the neighborhood-only model, there was a significant increase (p<0.05) in accuracy (=0.891), sensitivity (=0.931), NPV (=0.608), composite score (=3.123), and MCC (=0.561). However, this was accompanied by a significant (p<0.05) drop in specificity (=0.643). There was no significant change in PPV, though.

A ranked list of the top 50 neighborhood sequences and descriptive genomic features used to train NBDriver is shown in Table S4. Out of those 50 features, 26 were neighborhood-based features or the TF-IDF scores of the overlapping 4-mers extracted from a window size of 10. The plot displaying the variation in the AUROC with various classification thresholds is shown in Figure S3. The best results were obtained using a threshold of 0.119. Consequently, all mutations with the prediction scores above this threshold were classified as drivers and vice versa.

Overall, on this benchmarking dataset, NBDriver ranked fourth in terms of the composite score and MCC, fifth in terms of specificity and PPV and second in NPV, sensitivity, and accuracy. Both the composite score and MCC are indicative of balanced performance values for each of the classification metrics. Furthermore, there was no significant difference (*p* < 0.05) between NBDriver and CHASMplus in terms of MCC and composite score (Table 2; Table S5B). Compared to NBDriver, although the neighborhood-only model was the top-ranking tool in terms of specificity and PPV, it did not perform well in terms of the other metrics. Owing to NBDriver’s superior performance, all subsequent external validations were performed using this model.

### 3.4. Voting Ensemble of Prediction Algorithms Gives Better Classification Performances

We also assessed the effect of combining multiple top-ranked single predictors into an ensemble model. We evaluated NBDriver’s contribution to the overall ensemble by obtaining predictions without the tool. The top-performing ensemble (Ensemble 1) derived using the majority voting rule consisted of NBDriver, FATHMM (cancer), Condel, and MutationTaster. This resulted in a composite score of 3.583, sensitivity of 0.9953, MCC of 0.782, accuracy of 0.951, and NPV of 0.9596, significantly higher (p<0.05) than every single predictor evaluated in the study (Table 3; Table S5C). The composite score, MCC, NPV, sensitivity, and accuracy obtained using this ensemble were also the highest among all the different combinations of single predictors tested in this study (Table S5A,C). Removing NBDriver from the ensemble resulted in a significant decrease (p<0.05) in the composite score, NPV, MCC, accuracy, and specificity. However, it was accompanied by no significant change in PPV and sensitivity for the smaller ensemble (Table 3).

Another ensemble model (Ensemble 2), with a given mutation being considered a driver if at least two out of the four predictors called it a driver, consisted of NBDriver, CHASMplus, FATHMM (cancer), and MutationTaster (Table 3; Table S5A). This ensemble provided a significantly lower composite score of 3.522, NPV of 0.92, and an MCC of 0.75 as compared to Ensemble 1. However, there was no significant difference in PPV, specificity sensitivity, and accuracy (Table 3; Table S5A).

A complete set of all the different combinations of the single predictors evaluated in this study using the Martelotto et al. [45] dataset is present in Table S5A–C. From this table, we observed that out of all the ensemble algorithms tested, the maximum specificity (=0.9071) was obtained by the ensemble (MutationTaster, NBDriver, FATHMM(cancer), and CHASMplus). This predictor combination called a given variant driver if all four of MutationTaster, NBDriver, FATHMM(cancer), and CHASMplus called it a driver. On removing NBDriver from this ensemble, there was a significant decrease in the specificity (=0.8857). However, the maximum PPV (=0.9804) was obtained using the ensemble (NBDriver, CHASMplus, MutationTaster) (Table S5A). This predictor combination called a given variant driver if all three of MutationTaster, NBDriver, and CHASMplus called it a driver. Removing NBDriver from this ensemble, however, did not result in any significant change in PPV.

### 3.5. Driver and Passenger Mutations’ Features Used to Train NBDriver Are Significantly Different

**Figure 4:**
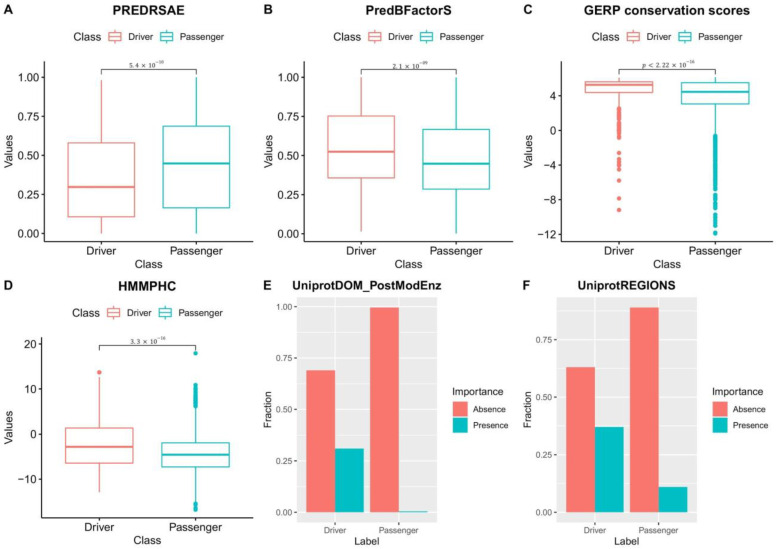
Class-wise distributional differences in features: Differences in the distribution of features between driver and passenger mutations observed from the training data used to derive NBDriver. (**A**) PREDRSAE (predicted residue solvent accessibility—exposed) gives the probability of the wild-type residue being exposed. From the plot, it is clear that the probability of driver mutations occurring in residues that are exposed is significantly less (Wilcoxon test; $p=5.4\times 10^{-10}$) than that of passengers. (**B**) PredBFactorS (high predicted Bfactor) gives the probability that the wild-type residue backbone is stiff. From the plot, it is clear that the probability of driver mutations occurring in residues with stiff backbones is significantly higher (Wilcoxon test; $p=2.1\times 10^{-9}$) than that of passengers. (**C**) GERP conservation scores provide the evolutionary conservativeness scores for specific sites where mutations have occurred. From the plot, it is clear that driver mutations occur in sites with GERP scores that are significantly higher (Wilcoxon test; $p<2.22\times 10^{-16}$) than passenger mutations. (**D**) HMMPHC (positional hidden Markov model (HMM) conservation score) is a measure that is calculated on the basis of the degree of conservation of the residue, the mutation, and the most probable amino acid. From the plot, it is clear that driver mutations tend to occur in residues with HMMPHC scores significantly higher (Wilcoxon test; $p=3.3\times 10^{-16}$) than passenger mutations. (**E**) UniprotDOM_PostModEnz is a feature based on protein domain knowledge that tells us whether a site in an enzymatic domain is responsible for any kind of post-translational modification (or PTM). Presence indicates that the mutation occurs in a site responsible for PTM and vice versa. From the plot, it is clear that more driver mutations occur in PTM-associated sites as compared to passengers. (**F**) UniprotREGIONS is a binary variable that tells us whether a mutation occurs in a region of interest in the protein sequence. Presence indicates that the mutation occurs in a region of interest and vice versa. From the plot, it is clear that more driver mutations cluster in regions of interest in the protein sequence than passengers, thereby making them mechanistically influential for the progression of the disease.

Our feature selection results from the external validation experiments reaffirm the observations made by Mao et al. [27] in terms of the differences in the underlying biological processes governing driver and passenger mutations. Using the training data used to build NBDriver, we found that driver mutations tend to occur on amino acid residues that have stiff backbones and have less solvent accessibility as denoted by the significantly lower (Wilcoxon test; $p<5.4\times 10^{-10}$) PREDRSAE probability measure (Figure 4A) and the significantly higher (Wilcoxon test; $p<2.1\times 10^{-9}$) ‘PredBFactorS’ probability measure (Figure 4B) respectively. We also observed that a mutation is more likely to be a driver if it occurs in genomic regions that were evolutionarily conserved. The mean GERP score for driver mutations was significantly higher (Wilcoxon test; $p<2.2\times 10^{-16}$) than that of passengers (Figure 4C). Similarly, driver mutations were more common in genomic sites that had a significantly higher (Wilcoxon test; $p<3.3\times 10^{-16}$) positional hidden Markov model (HMM) conservation score (or HMMPHC) as compared to passengers (Figure 4D). Among the other features, we observed similar class-wise distributional differences among features indicative of protein domain knowledge. UniprotDOM_PostModEnz denotes the presence or absence of a mutation in a site within an enzymatic domain responsible for post-translational modification (or PTM). PTM-related mutations are often accountable for changes in protein functions and alterations of regulatory pathways, eventually leading to carcinogenesis. UniprotREGIONS is another binary feature that tells us whether a mutation occurred in an experimentally defined region of interest in the protein sequence, such as those associated with protein–protein interactions and regulation of biological processes. Our analysis pointed out that a considerable portion (31%) of driver mutations clustered around PTM sites, contrasted by around 0.4% of passengers (Figure 4E). Similarly, about 37% of driver mutations were located in protein domains that were experimentally defined as regions of interest compared to around 11% of passengers (Figure 4F). Mao et al. [27] observed similar distributional differences among features such as the UniprotDOM_PostModEnz, UniprotREGIONS, GERP, PREDRSAE, and PredBFcatorS while trying to derive a cancer type-specific mutation effect prediction tool. They concluded that these results underscore the similarities and dissimilarities between the different cancer types and are indicative of the underlying mutagenic mechanisms.

In our approach, the TF-IDF algorithm was used to weigh a *k*-mer and assign importance to it in the given set of neighborhood sequences. In addition, a higher TF-IDF score is indicative of the greater relevance/importance of that *k*-mer. Our feature selection results indicated that for the 26 neighborhood sequence-based features, the mean TF-IDF scores for drivers were significantly higher (Wilcoxon test; p<0.05) than that of passengers (Figure S4). This result suggested that NBDriver’s top neighborhood features are more specific to the driver neighborhoods than the passengers.

### 3.6. Evaluation Using Previously Unseen Coding Mutation Data

To evaluate NBDriver’s capability at identifying previously unseen driver mutations, we evaluated it using missense mutation data compiled from the following four databases. We also reported the performances of the combination of the top four mutation effect predictors (based on the composite score) from Table 2.

### 3.7. Cancer Mutation Census

Based on the various evidence criteria set forth by the Cancer Mutation Census database, a particular mutation can be classified into tier 1, 2, or 3, with tier 1 mutations having the highest level of evidence of being a driver and so on. From the list of missense mutations in the CMC not present in our training data, NBDriver could accurately predict all 19 tier 1, 25 out of 28 tier 2, and 179 out of 230 tier 3 mutations, achieving an overall accuracy of 80.5% (Table S5D). On the other hand, the best ensemble performance was obtained using a combination of NBDriver and MutationTaster. This predictor combination called a given mutation driver if at least one of NBDriver and MutationTaster called it a driver, and it could accurately predict all 19 tier 1, 27 out of 28 tier 2, and 228 out of 230 tier 3 mutations achieving an overall accuracy of 98.91% (Table S5D). After removing NBDriver, the smaller ensemble consisting of just MutationTaster could accurately identify all 19 tier 1, 27 out of 28 tier 2, and 214 out of 230 tier 3 mutations resulting in a significantly (*p* < 0.05) reduced accuracy of 93.86% (Table S5D). Another ensemble model consisting of NBDriver and FATHMM(cancer) could accurately identify all 19 tier 1, 27 out of 28 tier 2, and 218 out of 230 tier 3 mutations resulting in an overall accuracy of 95.3%. Without NBDriver, the performance of the reduced ensemble consisting of only FATHMM(cancer) significantly (*p* < 0.05) dropped to 76.8% (Table S5D). Upon further investigation, we found that NBDriver was highly successful in identifying hotspot mutations present in the CMC. Recurrent alterations at the same genomic site in cancer genes such as MET, MPL, FLT3, and KIT have been implicated in many different cancer types [56,57,58,59,60,61] (Table S7A).

### 3.8. Cancer Genome Interpreter Database

Using pathogenic mutations compiled from various sources, we found that NBDriver could accurately identify 1274 out of 1628 non-overlapping missense driver mutations, achieving an overall accuracy of 78%. The model correctly identified all three mutations from the Cancer Biomarkers Database, 39 out of 47 mutations from the DoCM database, 23 out of 31 mutations from the Martelotto et al. study [45], and 1209 out of 1547 mutations from the OncoKB database. On the other hand, the best ensemble performance was obtained using a combination of NBDriver, CHASMplus, FATHMM (cancer), and MutationTaster. This predictor combination called a given mutation driver if at least one of the four predictors called it a driver, and it could accurately predict 1625 out of 1628 mutations achieving an overall accuracy of 99.81% (Table S5E). After removing NBDriver, the smaller ensemble consisting of MutationTaster, CHASMplus, and FATHMM (cancer) could accurately identify 1616 out of 1628 mutations resulting in reduced accuracy of 99.26% and did not significantly differ from the original ensemble (Table S5E). Another ensemble model consisting of CHASMplus, FATHMM(cancer), and NBDriver could accurately identify 1544 out of 1628 mutations resulting in an accuracy of 94.84%. The smaller ensemble, after removing NBDriver, correctly identified 1355 out of 1628 mutations resulting in a significantly (*p* < 0.05) reduced accuracy of 83.23% (Table S5E).

### 3.9. Recurrent Driver Mutations

Out of the top 33 hotspot mutations identified in the study conducted by Rheinbay et al. [48] as recurrently mutated, NBDriver correctly identified 27 as drivers. However, MutationTaster and CHASMplus displayed superior performance by identifying all 33 mutations correctly. On the other hand, FATHMM(cancer) identified 31 out of 33 mutations correctly, resulting in an accuracy of 93.9% (Table S5F). An ensemble consisting of NBDriver and FATHMM(cancer) could accurately identify all 33 mutations resulting in a significant (*p* < 0.05) increase in the accuracy (Table S5F). Except for KRAS, NBDriver correctly identified all mutations from the other four genes (NRAS, TP53, PIK3CA, and IDH1) as cancer drivers. Hotspot mutations in these four genes reported by Rheinbay et al. [48], correctly identified as drivers by NBDriver, have been implicated in many different cancers [62,63,64,65] (Table S7A).

### 3.10. Rare Driver Mutations Found in Glioblastoma and Ovarian Cancer

Using the list of rare drivers reported by the developers of the driver prediction tool CanDrA [27], we evaluated NBDriver’s ability to identify less frequent alterations in the cancer genome. Overall, NBDriver alone could identify 29 out of 34 (85%) glioblastoma mutations and 20 out of 38 (53%) ovarian cancer mutations. All these mutations belonged to eight known OVC-related genes (ARID1A, CDK12, ERBB2, MLH1, MSH2, MSH6, PIK3R1, PMS2) and seven known GBM-related genes (ATM, EGFR, MDM2, NF1, PDGFRA, PIK3CA, ROS1). All eight OVC-related genes correctly identified as drivers by NBDriver have been implicated in ovarian cancer through observations made from multiple studies [66,67,68,69,70] (Table S7B). The ensemble model consisting of NBDriver, FATHMM (cancer), and MutationTaster identified all 34 glioblastoma mutations and 36 out of 38 ovarian cancer mutations resulting in an overall accuracy of 0.9722 (Table S5G). However, no significant difference was observed in the accuracy after removing NBDriver from the ensemble. Another ensemble model consisting of NBDriver and MutationTaster could accurately identify 34 glioblastoma mutations and 34 ovarian cancer mutations resulting in an overall accuracy of 94.4%, whereas MutationTaster alone could identify 30 glioblastoma and 30 ovarian cancer mutations resulting in a significantly (*p* < 0.05) reduced accuracy of 83.33% (Table S5G).

### 3.11. Stratification of the Predicted Driver Genes Based on Literature Evidence

We combined the list of genes with at least one true positive missense driver mutation prediction from NBDriver into a catalog of 138 putative driver genes. We then compared our gene set against those already published in six landmark pan-cancer studies for driver gene identification. Bailey et al. [71] identified 299 driver genes from 9423 tumor exomes by combining the predictions from 26 different computational tools. Martincorena et al. [72] used the normalized ratio of non-synonymous to synonymous mutations (dN/dS model) to identify driver genes from 7664 tumors and reported a total of 180 putatively positively selected driver genes and 369 known cancer genes from three main sources:

(1) A total of 174 cancer genes from version 73 of the COSMIC database [6];
(2) A total of 214 significantly mutated genes across 4742 tumors identified by Lawrence et al. [73] using the MutSigCV tool;
(3) A total of 204 genes identified through a literature search.

Two marker papers from TCGA [74,75] identified 132 significantly mutated genes using the MutSigCV tool. Tamborero et al. [35] identified a list of 291 high-confidence drivers from 3205 tumor samples using a rule-based approach. Deitlein et al. [37] modeled the nucleotide context around driver mutations and identified 460 driver genes based on nucleotide context. Apart from the aforementioned studies, the overlap between our list of genes and two well-established cancer gene repositories: the Cancer Gene Census [6,76] and the Intogen database [77] was also reported. We identified 124 (=89%) of our predicted driver genes as canonical cancer genes present in the Cancer Gene Census. Among the remaining genes, six were cataloged as drivers in at least two of the pan-cancer studies or mutation databases as mentioned above (Table S6). A total of eight genes (*CTLA4*, *IGF1R*, *PIK3CD*, *TGFBR1*, *RAD54L*, *SHOC2*, *CDKN2B*, and *XRCC2*) were not identifiable from any of the landmark studies or databases and required further validation.

## 4. Discussion

Our investigation aimed to compare the raw neighborhood sequences of driver and passenger mutations and exploit any observed distributional differences to build robust classification models. Using generative models, we showed that except for one window size (*n* = 1), a significant difference in the distributions between the neighborhoods of driver and passenger mutations was consistently present in our cohort. Next, using the TF-IDF and count vectorizer scores derived from the overlapping *k*-mers, we trained a KDE-based generative classifier and two other tree-based classifiers. One crucial distinction between NBDriver and other methods is the inclusion of overlapping *k*-mers extracted from the neighborhood of mutations as features for further analysis. NBDriver was trained using a small set (=50) of highly discriminative features, 52% of which were neighborhood scores. Using this model, we could accurately predict 89% of all the literature-curated mutations outlined in the Martelotto et al. study [45], 81% of the high-confidence list of mutations recently published by the Cancer Mutation Census, 78% of all the actionable alterations reported in the Cancer Genome Interpreter, 82% of all the hotspot mutations reported from pan-cancer genome analysis, 85% and 53% of rare driver mutations found in glioblastoma and ovarian cancer, respectively. Ensemble models obtained by combining the predictions from other state-of-the-art mutation effect predictors with NBDriver performed significantly better than the individual predictors in all five validation datasets. These results underscore the importance of including neighborhood features to build mutation effect prediction algorithms.

We validated the true positive mutations that NBDriver identified with existing literature (Table S7A). The predicted driver mutations from the CMC have been implicated in many different types of cancers. For instance, mutations such as Y1248C and M1268I occur in the proto-oncogene MET and are associated with poor prognosis in renal cell carcinoma [57]. Similarly, the W515L mutations in the MPL oncogene are helpful in identifying patients with chronic myeloproliferative neoplasms [56]. Hotspot mutations occurring in the codon 835 of the FLT3 oncogene have been implicated in the majority of AML and ALL patients [59], and recurrent aberrations such as D816V and V560D found in the KIT oncogene have been associated with patients suffering from AML and gastric cancer, respectively [60,61]. Among the hotspot mutations from Rheinbay et al., NBDriver correctly identified mutations in the codon 1047, which are some of the most frequent alterations in the PIK3CA gene [64], mutations in the codon Q61, which is a predominantly mutated hotspot in NRAS implicated in melanoma [62], and evolutionarily conserved residue R132 in IDH1, which was found to be mutated in GBM [65].

Although our method’s focus was to identify missense driver mutations from sequenced cancer genomes, the majority of the genes (130 out of 138) containing at least one predicted mutation belonged to the Cancer Gene Census or other large-scale driver gene discovery studies. The protein products of the eight remaining genes not flagged as drivers by any of the databases/studies had known functional roles in maintaining the cancer genome’s stability and promoting tumor development. The CTLA4 gene modulates immune response by serving as checkpoints for T-cell activation, essentially decreasing the T cells’ ability to attack cancer cells. Immune checkpoint inhibitors, which are designed to “block” these checkpoints, have drastically changed the treatment outcomes for several cancers [78]. Transcriptomic profiling of blood samples drawn from cervical cancer patients identified IGF1R as a biomarker for increased risk of treatment failure [79]. Overexpression of the PIK3CD gene has been associated with cell proliferation in colon cancer and is responsible for poor prognosis among patients [80]. Multiple studies have indicated an association with polymorphisms observed in TGFBR1 and cancer susceptibility [81,82]. Similarly, polymorphisms detected in the RAD54L are a genetic marker associated with meningeal tumors’ development [83]. SHOC2 has been reported to be a regulator of the Ras signaling pathway and is associated with poor prognosis among breast cancer patients [84]. Similarly, the inactivation of the CDKN2B gene is responsible for the progression of pancreatic cancer [85]. With the help of massively parallel sequencing studies, rare mutations in the XRCC2 gene have been linked to increased breast cancer susceptibility among patients [86].

Our study does have some limitations. First, we used a representative dataset of driver and passenger mutations whose labels were not in silico predictions from other mutation effect prediction algorithms but derived from experimentally validated functional and transforming impacts from various sources. This resulted in a relatively small sample size for supervised classification. However, this approach also minimized the chances of inadvertently introducing false-positive mutations into the training set used to derive the driver and passenger neighborhoods’ class-wise density estimates or the machine learning models. Evidence [87] suggests that a sizable proportion of mutations present in large mutational databases are mostly false positives, reflecting sequencing errors due to DNA damage. Moreover, NBDriver derived using this high-confidence list of mutations performed reasonably well across all five independent validation sets and produced 138 driver genes with sufficient literature evidence suggesting that our initial choice of the training dataset was overall beneficial. Second, since missense mutations are the most abundant form of somatic alterations [88], our machine learning models were all trained using missense mutations only. However, in principle, our approach could be extended to other types of mutations as well.

Additionally, during the external validation analysis, although NBDriver performed very well in terms of PPV (=0.941), the NPV (=0.608) was relatively low (Table 2). To identify biologically relevant mutations for further functional validation, NPV is often overlooked as a classification metric. A high NPV allows us to exclude passenger mutations with greater confidence and reduces the number of driver mutations incorrectly labeled as passengers (false negatives). However, we observed that adding different combinations of multiple single predictors into ensemble models resulted in a significant improvement in the NPV (Table 3). Our observations on the ensemble models’ performances were similar to those made by Martelotto et al. [45]. Last, we trained our machine learning models using the combined dataset containing mutational effects determined from experimental assays not specific to any cancer type. Hence, all our models were pan-cancer-based. Consequently, a cancer type-specific analysis in the future would require the list of known driver and passenger mutations from specific tumor types.

## 5. Conclusions

In this study, we showed that there is a significant difference in the nucleotide contexts surrounding driver and passenger mutations obtained from sequenced cancer genomes. Using efficient feature representation, we generated robust classification models that provided comparable performances across five independent validation sets. The predicted true positive mutations were part of genes with experimental support of being functionally relevant from multiple sources. Future experiments using a much larger sample size need to be performed to derive neighborhood-sequence-based classification scores for all possible missense mutations in the genome across several cancer types. This would be possible if future large-scale sequencing studies such as MSK-IMPACT [89], PCAWG [48], ICGC [7], and GENIE [90] produce a complete catalog of missense driver mutations with functional evidence in a cancer type-specific manner. This relatively novel strategy of using the sequence neighborhoods for driver mutation identification can dramatically improve the annotation process’s efficiency for unknown mutations.

## Figures and Tables

**Table 1:** Median JS distances for both the original and randomized experiments for different window sizes.

Window Size	Feature Type	Median JS Distance (Original)	Median JS Distance (Randomized)	*p*-Value
1	TF (*k* = 2)	0.345	0.339	Not significant
2	OHE	0.275	0.228	<0.05
3	CV (*k* = 2)	0.219	0.177	<0.05
4	TF (*k* = 3)	0.214	0.167	<0.05
5	CV (*k* = 3)	0.211	0.166	<0.05
6	TF (*k* = 4)	0.210	0.166	<0.05
7	CV (*k* = 2)	0.211	0.165	<0.05
8	TF (*k* = 3)	0.211	0.164	<0.05
9	TF *(k* = 3)	0.211	0.166	<0.05
10	TF (*k* = 4)	0.211	0.165	<0.05

**Table 2:** Comparison of the generated binary classifiers with other mutation effect prediction algorithms using the benchmarking dataset published by Martelotto et al. (Ranked in decreasing order on the basis of the composite score or CS).

Algorithm	Accuracy	Sensitivity	Specificity	PPV	NPV	CS	MCC
MutationTaster	0.8857	0.9081	0.75	0.9566	0.5738	3.1885	0.590
FATHMM (Cancer)	0.91	0.9788	0.4929	0.9213	0.7931	3.1861	0.580
CHASMplus (Pancancer)	0.85	0.852	0.85	0.972	0.486	3.16	0.570
**NBDriver**	**0.891**	**0.931**	**0.643**	**0.941**	**0.608**	**3.123**	**0.561**
**Neighborhood-only model**	**0.85**	**0.629**	**0.907**	**0.9744**	**0.285**	**2.7954**	**0.370**
Condel	0.8584	0.9258	0.45	0.9108	0.5	2.7866	0.392
FATHMM (missense)	0.8251	0.8775	0.5071	0.9152	0.4057	2.7055	0.351
PROVEAN	0.7371	0.7444	0.6929	0.9363	0.3089	2.6825	0.327
SIFT	0.8099	0.861	0.5	0.9126	0.3723	2.6459	0.32
Polyphen-2	0.7978	0.8422	0.5286	0.9155	0.3558	2.6421	0.317
Mutation Assessor	0.747	0.7665	0.6286	0.9259	0.3077	2.6287	0.3
VEST	0.7503	0.8269	0.2857	0.8753	0.2139	2.2018	0.1
CanDrAplus (Cancer-in-general)	0.592	0.857	0	0.99	0	1.847	−0.03

The bold format numbers represent the results obtained from the two binary classifiers trained using the neighborhood features.

**Table 3:** Evaluating the contribution of NBDriver to the top-performing ensemble predictors.

Ensemble ID	Algorithm	Accuracy	Sensitivity	Specificity	PPV	NPV	CS	MCC
Ensemble 1 (With NBDriver)	**NBDriver** + FATHMM (cancer) + MutationTaster + Condel	0.9505	0.9953	0.6785	0.9494	0.9596	3.583	0.782
Ensemble 1 (Without NBDriver)	FATHMM (cancer) + MutationTaster + Condel	0.917	0.9941	0.5357	0.9285	0.9375	3.395	0.677
Ensemble 2 (With NBDriver)	**NBDriver** + CHASMplus + FATHMM (cancer) + MutationTaster	0.948	0.991	0.664	0.947	0.92	3.522	0.754
Ensemble 2 (Without NBDriver)	CHASMplus + FATHMM (cancer) + MutationTaster	0.917	0.939	0.756	0.963	0.679	3.367	0.682

## Data Availability

Publicly available datasets were analyzed in this study. The list of mutations from the Brown et al. [39] study was downloaded from the Supplementary Materials published as part of the study https://journals.plos.org/ploscompbiol/article?id=10.1371/journal.pcbi.1006981#sec020 (accessed on 2 November 2019). The list of mutations from the Martelotto et al. [45] benchmarking study was downloaded from the “Additional file 2” of the Supplementary Materials published as part of the study https://genomebiology.biomedcentral.com/articles/10.1186/s13059-014-0484-1#Sec20 (accessed on 2 November 2019). The Cancer Mutation Census data was downloaded from https://cancer.sanger.ac.uk/cmc/home (accessed on 3 October 2020). The Catalogue of Validated Oncogenic Mutations was downloaded from https://www.cancergenomeinterpreter.org/mutations (accessed on 15 November 2020). Rare driver mutations from the driver prediction tool CanDrA [27] were downloaded from the Supplementary Tables S8 and S9 published as part of the study. The coding mutations from Rheinbay et al. [48] were downloaded from the “Extended Data Figure 1” published as part of this study https://www.biorxiv.org/content/10.1101/237313v1.full.pdf (accessed on 15 November 2020). GERP scores were obtained using the OpenCRAVAT web server (https://opencravat.org/, accessed on 15 November 2020), and all features from the SNVBOX were obtained using the hg19 version of the CRAVAT web server (http://hg19.cravat.us/CRAVAT/ (accessed on 30 October 2020). The KDE classifier class released as part of the “Python Data Science Handbook” by Jake VanderPlas [91] was used in our analysis to conduct the KDE-based classifications. All the codes necessary to reproduce the results of this manuscript are available at https://github.com/RamanLab/NBDriver (accessed on 18 February 2021).

## Supplementary Materials

The following are available online at https://www.mdpi.com/article/10.3390/cancers13102366/s1, Figure S1: Cross-validation framework, Figure S2: KDE workflow, Figure S3: Variation in AUROC with different classification thresholds, Figure S4: Class-wise variation in the mean TF-IDF scores for the neighborhood sequence features, Figure S5: Workflow for deriving NBDriver, Figure S6: Classification performances from the repeated cross-validation experiments, Table S1A: Details of the five datasets that were combined to form one single dataset of missense mutations in Brown et al., Table S1B: Summary of datasets used in this study, Table S2A: List of the descriptive genomic features used to train our machine learning models, Table S2B: Number of one-hot encoded features and possible k-mers for a given window size, Table S3A: Ranked (in descending order) classifier-feature combinations from the repeated cross-validation experiments using only neighborhood sequences as features, Table S3B: Results from the Wilcoxon signed-rank test displaying significant increase in the best median classification performances with the increase in the size of the neighborhood, Table S3C: Ranked median and randomized JS distances between the density estimates for all window sizes and seven different feature representations, Table S3D: Significance of increase in the ranked estimates shown in the Table S3C, Table S4: Ranked list of the 50 features used to train the integrated model, Table S5A: Classification results obtained on the Martelotto et al. dataset obtained using the combinations of different mutation effect predictors, Table S5B: Classification results on the Martelotto et al. dataset obtained using single predictors, Table S5C: Results of combining various mutation effect predictors on the Martelotto et al. dataset using the majority voting rule, Table S5D: Classification results on the Cancer Mutation Census dataset obtained using the combinations of different mutation effect predictors (with and without NBDriver), Table S5E: Classification results on the Cancer Genome Interpreter dataset obtained using the combinations of different mutation effect predictors (with and without NBDriver), Table S5F: Classification results on the Rheinbay et al. dataset obtained using the combinations of different mutation effect predictors (with and without NBDriver), Table S5G: Classification results on the Mao et al. dataset obtained using the combinations of different mutation effect predictors (with and without NBDriver), Table S6: List of driver genes predicted by our method and their overlap with gene sets from already published landmark studies, Table S7A: Summary and literature evidence of hotspot mutations correctly identified by our method, Table S7B: Literature evidence of the genes containing correctly predicted rare driver mutations by our integrated model.
