# Supplementary material for: Sequence Neighborhoods Enable Reliable Prediction of Pathogenic Mutations in Cancer Genomes
Source: Cancers (Basel). 2021 May 14;13(10):2366. doi: 10.3390/cancers13102366 (PMC8156421; doi:10.3390/cancers13102366)
Supplement: Supplementary file 1 [file cancers-13-02366-s001.zip › Supplementary Figures.pdf]

Sequence Neighborhoods Enable Reliable Prediction of Pathogenic Mutations in Cancer Genomes

Shayantan Banerjee, Karthik Raman and Balaraman Ravindran

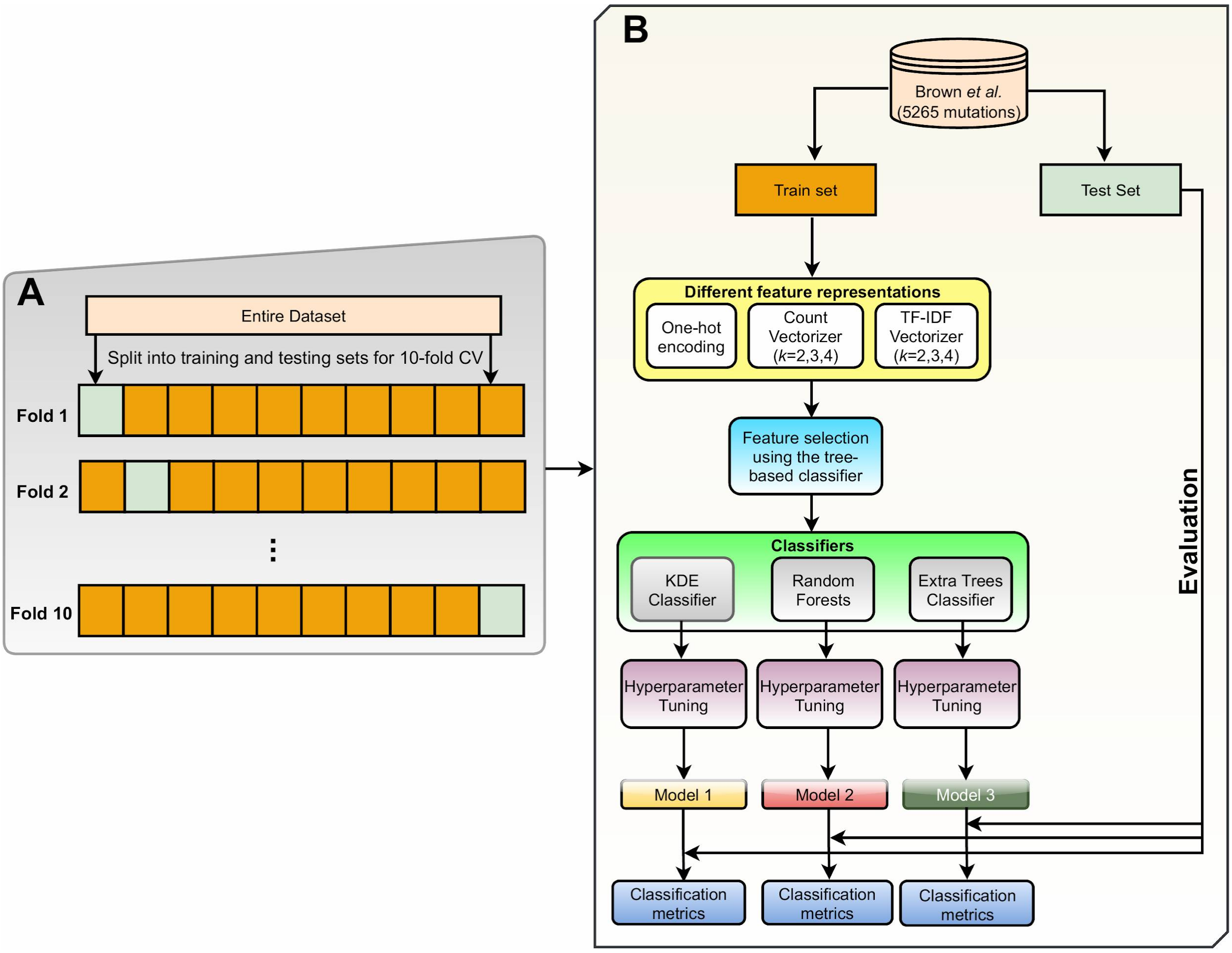

Figure S1. Cross-Validation framework: The workflow depicting one run of the 10-fold cross-validation experiments is shown in this figure. (**A**) In the first step, the entire dataset was split into ten equal parts. Nine of the ten subsets were combined into one training set, and one part was left as the test set. (**B**) Seven different feature representations [OHE, Count Vectorizer ($k = 2,3,4$) and TF-IDF Vectorizer ($k = 2,3,4$)] were considered for further analysis. After feature selection using a tree-based classifier, hyperparameter tuning was performed for three classifiers, and the corresponding models were derived. Finally, validation of each of the classifiers on the test set was performed, and the corresponding performance metrics were reported.

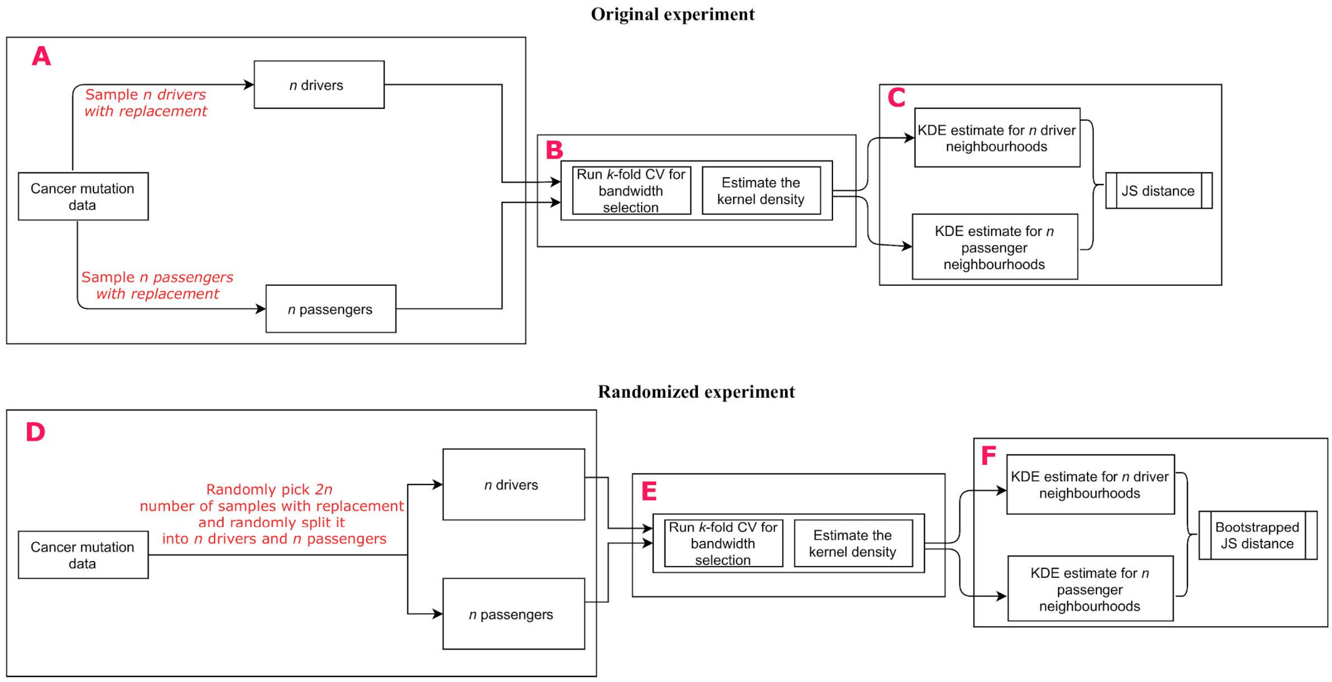

Figure S2. KDE workflow: The workflow depicting one run of the kernel density estimation experiment is shown in this figure. All 5265 mutations from the Brown et al. study were used to derive the estimates. (**A**) First, an equal number of driver and passenger mutations were sampled with replacement. (**B**) The “bandwidth” hyperparameter was tuned using a 5-fold cross-validation approach, and the resulting tuned hyperparameter was used to estimate the densities. (**C**) The kernel density estimates for the driver and passenger neighborhoods were obtained separately, and the distance between them was calculated using the Jensen-Shannon (JS) distance. The JS distance is used to quantify how “distinguishable” two probability distributions are from each other. It is bounded between 0 and 1, where 0 represents the case where the two probability distributions are equal and vice versa. (**D**) The bootstrapping experiment to compute the significance of the density estimates calculated in (C) is shown in this figure. First, it involved random sampling of twice the driver or passenger mutations from (A) irrespective of the labels, followed by randomly splitting the data into driver and passenger labels. (**E**) Hyperparameter tuning and density estimation was performed similarly to (B). (**F**) The bootstrapped JS distance between the driver and passenger neighborhoods was derived. All six steps (A–F) of the density estimation experiments were repeated 30 times for all possible window sizes between 1 and 10 and seven different feature representations. The significance of the difference between the medians of the original and the bootstrapped JS distances was then reported.

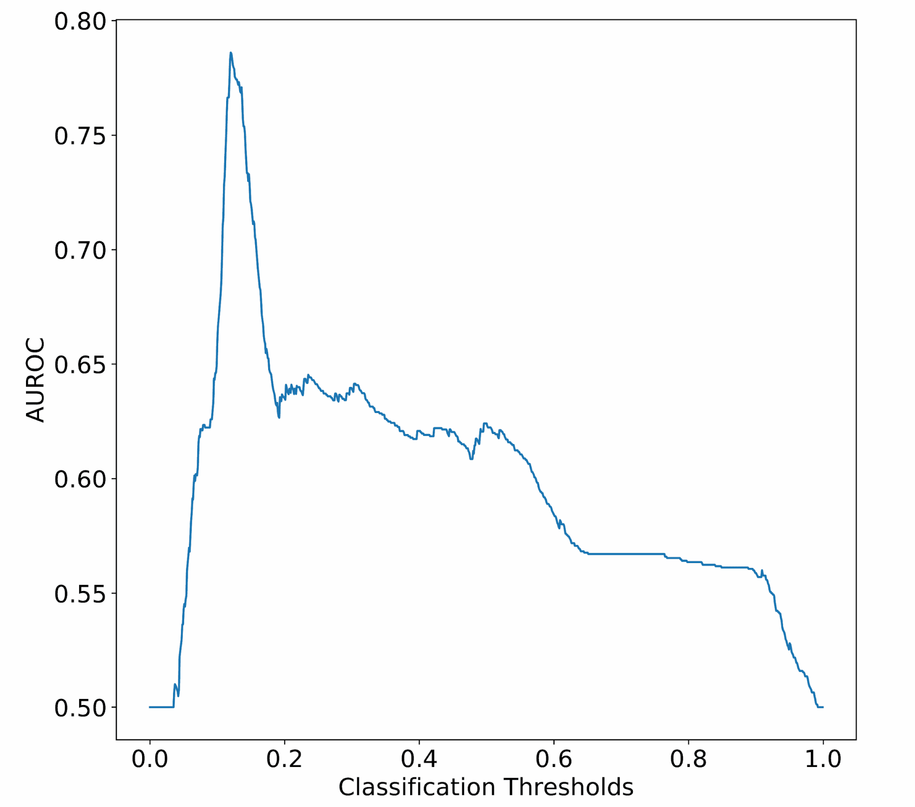

Figure S3. Variation in AUROC with different classification thresholds: Plot showing the variation in AUROC with the different classification thresholds obtained while deriving NBDriver is shown here. NBDriver was trained on a reduced training set of 4549 mutations after removing all overlapping mutations from the original study and Martelotto et al. For an imbalanced classification problem, using the default threshold of 0.5 is often not advisable. In our case, the best AUROC was obtained using a threshold of 0.119. Consequently, all mutations with prediction scores greater than this threshold were classified as drivers and vice versa.

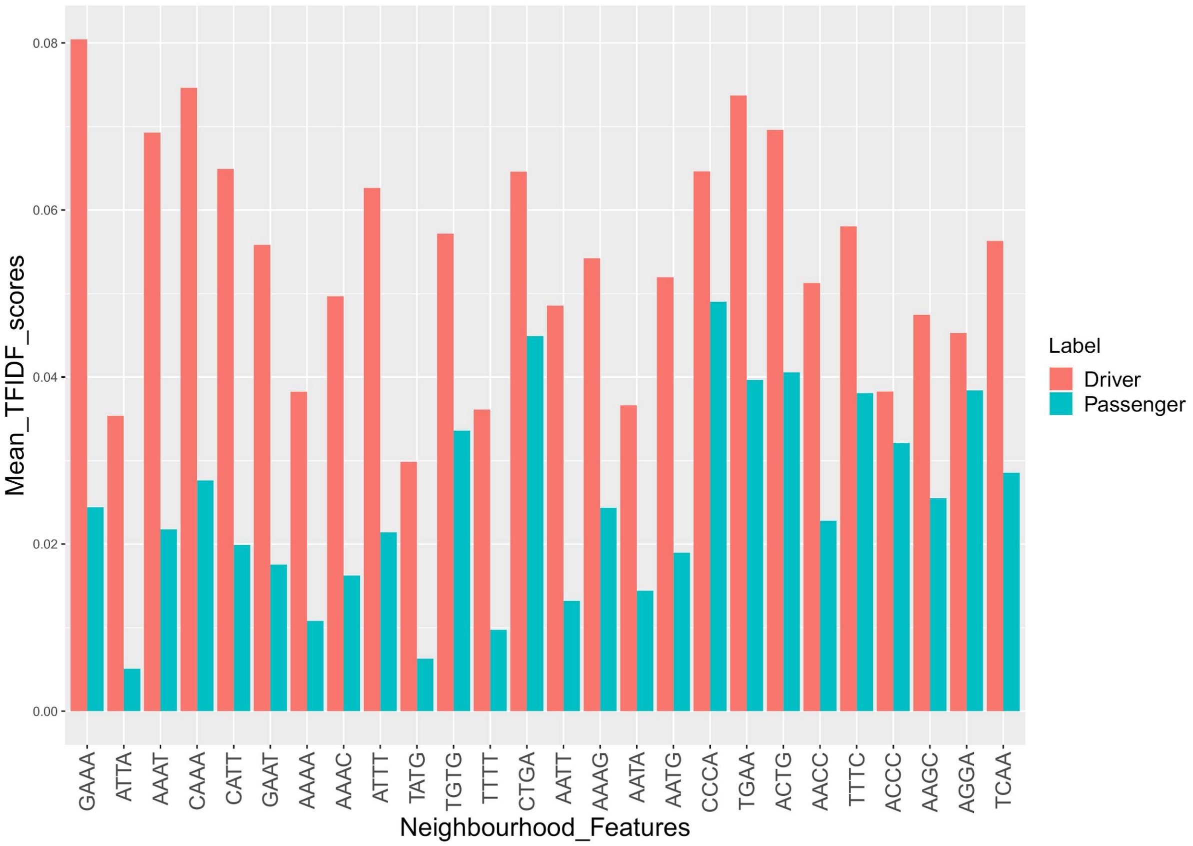

Figure S4. Class-wise variation in the mean TF-IDF scores for the neighborhood sequence features: Plot showing the class-wise variation in the mean TF-IDF scores for the 26 neighborhood-sequence features used to train NBDriver. The *x*-axis represents the 4-mers extracted from the neighborhood sequences, and the *y*-axis represents the mean TF-IDF scores. From the plot, it is evident that the mean TF-IDF scores are consistently higher for drivers as compared to passengers. Since a higher TF-IDF score indicates the relevance or importance of a particular *k*-mer, we can conclude that the 4-mers used to derive NBDriver are more specific to the driver neighborhoods than passengers.

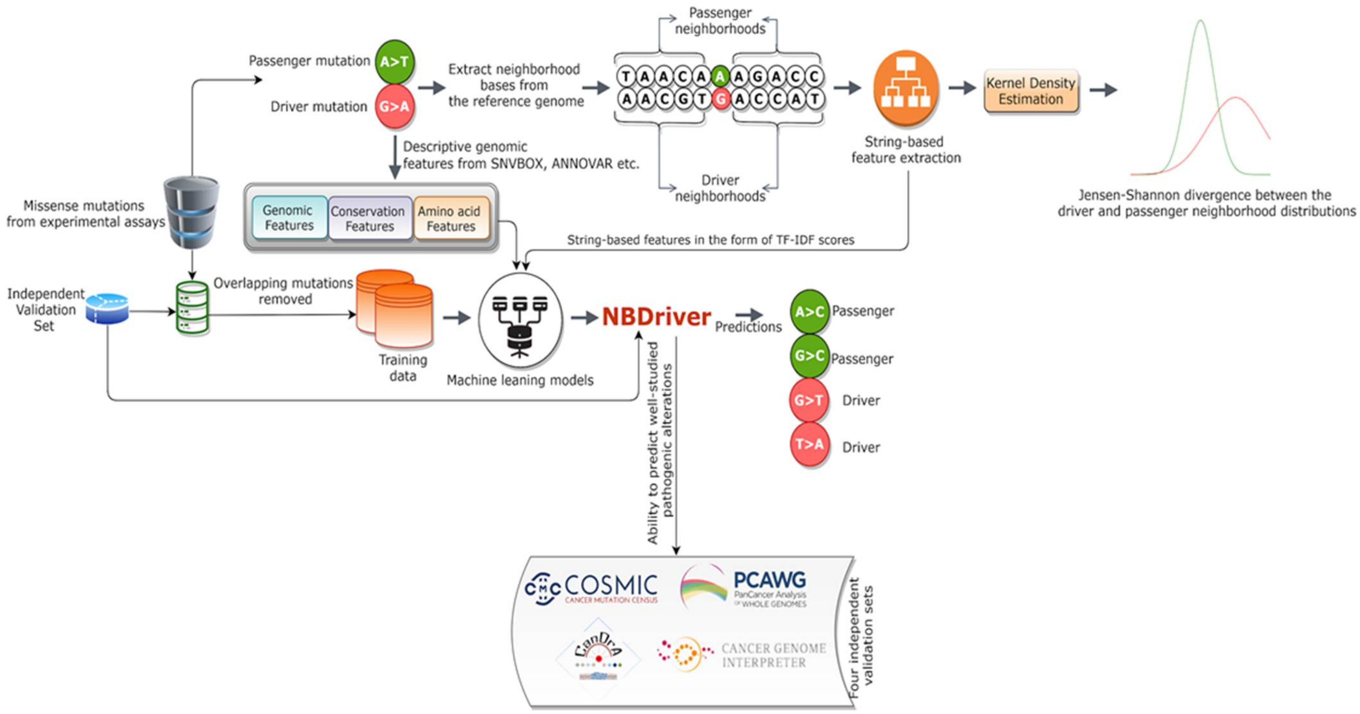

Figure S5. Workflow for deriving NBDriver: Overall workflow of our approach for delineating the distributional differences between driver and passenger neighborhoods and subsequent development and validation of the machine learning-based driver discovery tool-NBDriver.

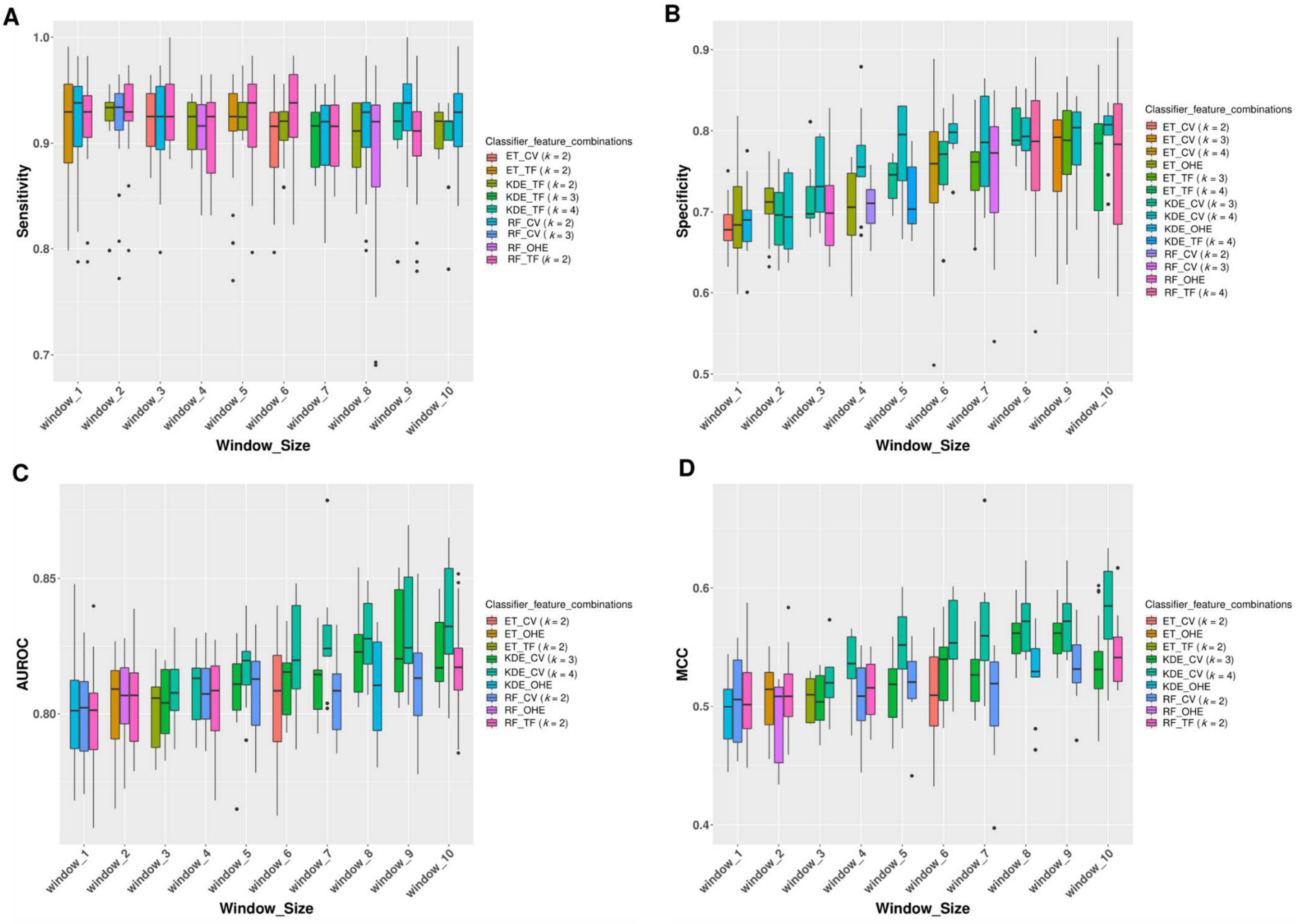

Figure S6. Classification performances from the repeated cross-validation experiments: Top three classifier-feature combinations based on the different classification metrics (**A**) sensitivity, (**B**) specificity, (**C**) AUROC, and (**D**) MCC obtained from the repeated cross-validation experiments is shown in this figure.
